# Rice

**Published:** 1885-03

**Authors:** 


### RICE.

Boiled rice is among the most nutritious of all foods, and is the most easily digested. Being very cheap in all countries it has become the principal food of more than half the human family.

In cold weather it is excellent food, for it gives to the human body four times as much heat as an equal weight of beef. It is digested quickly and easily, even by weak stomachs. While giving heat to the body, it lacks the elements which replace waste tissue, and consequently it is a poor food for one who expects to undergo violent exercise. Beef is in this respect about four times as efficient as rice.

In China rice is among the masses, almost the only food, and as they have used it longer and to a greater extent than any other nation, it is probable that their plan for cooking it is as near per fection as possible.

By the Chinese method it is cooked as follows: Take a clean stewpan with a close fitting cover, and a piece of muslin somewhat larger than the top of the pan, so that when placed over the top of the pan, it will hang down in the middle so as to nearly touch the bottom of the pan, and form a sort of bag. Put into this depression a half pint af rice, and pour over it a pint of water. Then put on the stove and boil slowly. Keep the cover on tightly. The water should not reach up far enough in the pan to touch the rice. In this way the rice will be steamed, and each grain will be perfect.

In China there is a peculiar custom of giving alms. Each store has a box of rice, from which a clerk gives the quantity that he can grasp between his thumb and finger, a mere pinch, to each beggar.

When one remembers how rice swells in cooking, the amount does not seem so small.

Beggars have regular routes from store to store, and it is said that they live well.

